# A framework for quality control in quantitative proteomics

**DOI:** 10.1101/2024.04.12.589318

**Published:** 2024-04-13

**Authors:** Kristine A. Tsantilas, Gennifer E. Merrihew, Julia E. Robbins, Richard S. Johnson, Jea Park, Deanna L. Plubell, Eric Huang, Michael Riffle, Vagisha Sharma, Brendan X. MacLean, Josh Eckels, Michael B. Bereman, Sandra E. Spencer, Andrew N. Hoofnagle, Michael J. MacCoss

**Affiliations:** 1 Department of Genome Sciences, University of Washington, Washington 98195, United States; 2 LabKey, 500 Union St #1000, Seattle, Washington 98101, United States; 3 Department of Biological Sciences, North Carolina State University, Raleigh, North Carolina 27607; 4 Canada’s Michael Smith Genome Sciences Centre (BC Cancer Research Institute), University of British Columbia, Vancouver, British Columbia V5Z 4S6, Canada; 5 Department of Laboratory Medicine and Pathology, University of Washington, Seattle, Washington 98195, United States

**Keywords:** quality control, proteomics, mass spectrometry, liquid chromatography, sample preparation, system suitability, quantitative results, PRM, DIA, DDA

## Abstract

A thorough evaluation of the quality, reproducibility, and variability of bottom-up proteomics data is necessary at every stage of a workflow from planning to analysis. We share real-world case studies applying adaptable quality control (QC) measures to assess sample preparation, system function, and quantitative analysis. System suitability samples are repeatedly measured longitudinally with targeted methods, and we share examples where they are used on three instrument platforms to identify severe system failures and track function over months to years. Internal QCs incorporated at protein and peptide-level allow our team to assess sample preparation issues and to differentiate system failures from sample-specific issues. External QC samples prepared alongside our experimental samples are used to verify the consistency and quantitative potential of our results during batch correction and normalization before assessing biological phenotypes. We combine these controls with rapid analysis using Skyline, longitudinal QC metrics using AutoQC, and server-based data deposition using PanoramaWeb. We propose that this integrated approach to QC be used as a starting point for groups to facilitate rapid quality control assessment to ensure that valuable instrument time is used to collect the best quality data possible.

## Introduction

Liquid chromatography coupled to tandem mass spectrometry (LC-MS/MS) is a sensitive and powerful approach to characterize the proteome using quantitative technologies. Historically, discovery experiments prioritized detecting the most peptides and proteins in a sample. The field has moved towards more complex and scaled-up applications that prioritize throughput and quantitation. Proteomics is increasingly relevant in the clinic in pursuit of disease diagnostics and novel biomarkers, and further development of stringent quality controls is necessary to propel this quantitative work going forward^1–4^. Despite best practices and best intentions, issues in sample processing and system function will occur, but the source of an issue is not always immediately clear, and prompt identification of problems is crucial for time and cost management.

In a typical bottom-up quantitative proteomics experiment, variability can be introduced from system function, sample preparation, and downstream analyses. Rapid, consistent, and longitudinally-tracked injections of reference samples to assess system suitability have been used to verify whether an LC-MS system is functioning adequately within desired tolerances. These runs effectively serve as a “canary in the coalmine” to provide early indications of an LC-MS system regression. Crucially, if we cannot obtain consistent peak areas, retention times, and mass accuracy, then quantitative experiments will be challenging. Beyond the system, much of the variability in quantitative studies originates from sample preparation^5^. This variability can include issues in protein extraction, digestion, and clean-up, and in sample batch effects. A strategy must be in place to assess the quality of the entire process including sample preparation, signal processing, normalization, and batch correction.

Any issues with the LC or MS impact data quality and challenge interpretation of results. If the system cannot generate reproducible measurements from equivalent quantities, any results collected thereafter are not reliable. Thus, rapid identification of these issues prior to and throughout sample data acquisition is crucial. Large experiments with runtimes on the scale of weeks to months will inevitably experience changes in instrument sensitivity or chromatography, therefore the ability to quantify the degree of change in repeated system suitability measurements throughout an experiment is useful. Early attempts to assess LC-MS systems in bottom-up proteomics focused on evaluating results such as the number of identified peptides post-database searching^6,7^. These identifications were used as a proxy for system function. However, this is complicated by the time requirements and potential variability introduced by database searches. We and others incorporated a statistical process control (SPC) into our bottom-up proteomics QC workflow (reviewed by Bramwell in 2013^8^ which often focuses on using identity-free metrics to track outputs over time relative to a baseline. The system suitability protocol developed by the Clinical Proteomic Tumor Analysis Consortium (CPTAC)^9^ to evaluate targeted proteomics assays across 11 institutions and 15 instruments used chromatographic and MS metrics including normalized peak area, chromatographic resolution, peak capacity, peak tailing, retention time drift, column conditioning and carryover. The Anubis/QCHtmlSummary platform used SRM to track longitudinal system function in peptides derived from a bovine protein mixture^10^. Our group incorporated SPC into SProCop and later automated SPC in Panorama AutoQC^11,12^. Many QC platforms have been developed in the past decade that incorporate longitudinal system monitoring and use multi-variable metrics to identify when the LC-MS system is not functioning optimally. To name just a fraction of this work spanning cloud-based applications, programming packages, or web interfaces includes SIMPATIQCO^13^, Metriculator^14^, iMonDB^15^, InSPECtor^16^, MSstatsQC^17^, QC-ART^18^, QCloud/QCloud2^19,20^, RawTools^21^, and Rapid QC-MS^22^. In addition to software tools, a stable and easy to obtain reference sample that can be measured repeatedly and reliably to assess system function is crucial. Such reference samples have been reported by numerous groups. Examples include the NIST reference material (RM) 8323 yeast protein extract^23^, a mixture of 6 bovine proteins^10^, and commercially-available human protein extract^24^.

In addition to instrument performance, sample processing reproducibility is invaluable to track. Sample processing is inherently variable due to differences in sample collection or storage^3,25,26^, digestion conditions^27^, enzyme efficiency^28,29^, contaminants^30^, and unidentified issues with reagents or protocols. It is crucial to identify these issues as they arise and ensure that any differences observed in the results are primarily due to biological variation rather than technical variation associated with sample preparation. Controls can be incorporated into individual samples (internal QCs), or additional samples can be prepared alongside experimental samples (external QCs). Early areas of focus in the development of proteomic reference materials and internal QCs involved the use of isotopically-labeled peptides for quantitation of specific proteins in targeted assays such as IS-PRM and QconCAT^31–34^. Different internal QCs can be introduced into a workflow at numerous stages to analyze more specific aspects of a process. For example, exogenous proteins such as lysozyme or ovalbumin have been used as process controls to evaluate sample preparation^35,36^. More recent efforts have focused on synthetic proteins that produce peptides which can be digested consistently and reproducibly, and span the chromatographic gradient. Additionally, others have developed protein internal QCs with derivative peptides that also function as indexed retention time (iRT)^37^ for scheduling or normalization such as DIGESTIF^38^ and RePLiCal^39^. Peptide internal controls added directly to samples have been used to assess system performance, normalize results, and as iRT standards^40,41^. Protein internal QCs added at the beginning of preparation allow examination of the full process from lysis, to digestion, to clean-up. Peptide internal QCs added just before MS analysis can be used to continuously monitor instrument function. When protein and peptide internal QCs are used together, issues in sample preparation can be distinguished from LC-MS system function problems. External QCs, when composed of a well-characterized matrix and prepared repeatedly within a batch and across an experiment, can identify issues in sample preparation. For example, a known mixture of phosphoproteins can be prepared as an additional sample to assess phosphopeptide enrichment efficiency^42^. Additionally, the use of external QC samples that are prepared multiple times within a batch can be used to assess the reproducibility and consistency of sample preparation. It is important to note that even when adequate QCs are in place to identify batch effects, additional steps prior to sample preparation and data collection are crucial to reduce the severity of batch effects that must be corrected. Especially in biologically-focused studies, the method and order in which samples are collected can be a significant driver of variation in quantitative experiments. To reduce the contribution of batch effects, it is essential to carefully and proactively plan sample batches that include blocking and randomization^43^ of experimental groups before processing. Approaches to reduce biases due to batch effects have been thoroughly reviewed by others^44^, and will not be reviewed in detail here.

Finally, once the system function and sample processing are found to be within expectations, the experimental results can be confidently considered. Many analytical tools have been developed to assess sample data quality. Early examples include the NIST MSQC pipeline^45^ and QuaMeter^46^ which often relied on identity-based metrics^46^. QuaMeter and other tools later expanded into identity-independent, multivariate analyses that can be applied to assess sample runs^18,47,48^. If the experiment goal is to maximize detections, identity-based metrics to determine data quality can be useful. However, in quantitative proteomics experiments these identity-based metrics no longer serve to adequately assess the system, sample preparation, or data quality. Identifications will change depending on the database searching scheme employed. Evaluating the consistency of metrics in raw, unnormalized data across runs, batches, and experiments is of greater utility. This can include metrics such as peak area, mass accuracy, isotopic distributions, and retention times of known quantities of the same analytes. To further improve data collection, QC steps may be implemented at the beginning of an experiment. Minimum steps to consider include planning for incorporation of QC samples beginning with experimental design and blocking samples representatively according to biological variables during sample collection and processing to reduce batch effects. After data collection, these QC metrics can aid in the selection of optimum normalization and batch correction methods. Many groups have developed effective ways to normalize sample data after database searching which has been reviewed by others^49^. Each sample matrix and MS collection scheme is unique, and the selection of normalization methods may change accordingly. For example, data-dependent acquisition methods suffer from missing data due to the irregular nature of sampling, which complicates analyses and normalization. Existing tools such as pmartR and the ProNorM workflow have endeavored to improve post-processing of datasets with missing data^50,51^. While we advocate for the use of peptide-level quantitation given the ambiguities associated with protein-level quantitation^52^, approaches to normalize data at the peptide and protein-level will differ. Crucially, determining whether normalization and batch correction in a data analysis pipeline has effectively reduced variance without nullifying biological differences can be challenging^53^. In addition to assessing the consistency of sample preparation, external QCs can be used to evaluate normalization and batch correction methods that are applied across experimental samples. External QCs are known reference samples that are prepared within each batch across an experiment and are processed from the start of an experiment through an entire protocol. Thus, assessing which normalization and batch correction methods reduce the technical variation in these samples can be a strong metric for improving confidence in quantitative approaches to analyzing bottom-up proteomics data.

**Figure 1:**
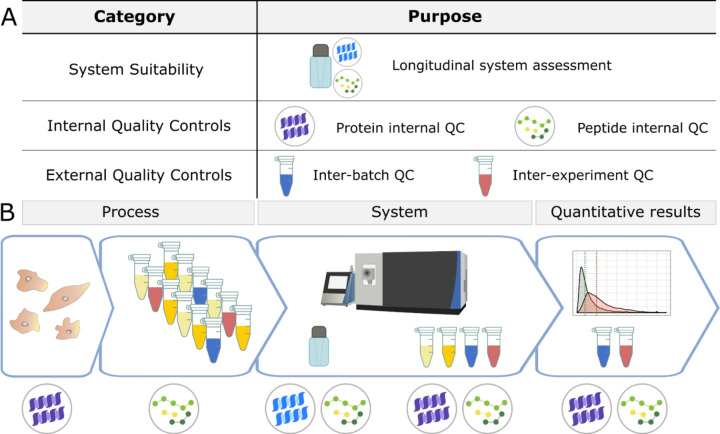
We use three categories of quality controls (QC) in our proteomics experiments: system suitability, internal QCs, and external QCs (A). The system suitability control is used to verify that the LC-MS system is functioning before and throughout sample data acquisition. Internal QCs are added to experimental samples to assess protein and peptide-level deviations in sample processing as well as instrument function. External QCs are additional samples prepared alongside experimental samples to monitor sample processing and batch effects. These samples are ideally formulated by pooling the experimental samples and are prepared multiple times within a batch. They also contain the same internal QCs used in experimental samples. External QCs can serve two functions: to assess the sample preparation workflow, and for normalization. In the context of a workflow (B), internal and external QCs must be planned for and incorporated beginning with sample collection and processing. Before performing any data analysis, the variance of internal and external QCs are examined. In combination, these controls can be used to evaluate sample processing and quantitative results within an experiment, as well as the LC-MS system function during an experiment and longitudinally over years.

Here, we present a customizable framework for QC in quantitative bottom-up proteomics experiments including real-world examples where this workflow was used to identify and resolve sample processing and instrumentation issues. We evaluate our quantitative proteomics experiments at three levels in 3 major stages: the system, the sample, and the entire workflow (Figure 1). Three classes of QCs (Figure 1) are implemented using three sample types: a study-independent system suitability standard, internal QCs, and a matrix-specific external QC. These controls differ in composition, purpose, preparation, and the stage they are introduced into a workflow, but all are used as “checkpoints” for quality assessment of quantitative experiments. Furthermore, we use a digital notebook to track maintenance and known issues, peaks are visualized in Skyline^54,55^, and system suitability MS data files are uploaded automatically to PanoramaWeb^56^ using Panorama AutoQC^12^. This adaptable approach to QC has allowed us to quickly identify problems in an experiment, pinpoint their origin, and improve quantitative outcomes by reducing the contribution of technical variation. We present examples that assessed diverse sample matrices including plasma, cerebrospinal fluid (CSF), yeast, brain tissue, and commercially-available reagents. We also discuss criteria for the selection and optimization of QC samples and reagents.

## Experimental Procedures

### Quality Control Sample Composition and Associated Reagents

Figure 1 illustrates the various QCs used (A) and the stages they are introduced (B). For our system suitability control, we typically inject 30–150 fmol Pierce^™^ Retention Time Calibration (PRTC) Mixture (Thermo Scientific) in a carrier background of 600 fmol of a bovine serum albumin (BSA) tryptic digest in 0.1% trifluoroacetic acid. For internal QCs, we typically begin by adding 16 ng yeast enolase per µg total protein in the sample (based on a BCA protein assay), which captures variation in the sample processing including tryptic digestion and LC-MS. A second internal QC (30–150 fmol per LC-MS run) of PRTC is added just prior to LC-MS analysis, which captures variation from the LC-MS steps. The quantity of peptide internal QCs are kept consistent between the system suitability and experimental samples. We use two forms of external QC samples: one to assess sample preparation, and one for normalization or batch correction. The external QC samples can be composed of two of the following options, with the best options listed first: a pool derived from representative experimental samples, additional samples from the same source as the experimental samples, or the same type of sample but from another source. The same protein and peptide internal QCs should be included in the external QC samples.

### Example 1: System Suitability on Orbitrap Eclipse Tribrid

The system suitability standard was loaded onto Kasil1 (PQ Corporation) fritted microcapillary trap (2–3 cm x 150 µm) loaded with 3 µm Reprosil-Pur C18 (Dr. Maisch) reverse-phase beads. Once the sample was loaded to the trap, it was brought inline with a 30 cm x 75 µm packed tip (New Objective) containing the same resin used for the trap. The high-performance liquid chromatography (HPLC) was a Thermo Easy nLC1200 and used 0.1% formic acid in water as buffer A, and 0.1% formic acid in 80% acetonitrile as buffer B. The strong needle wash was 50% acetonitrile, and the weak needle was 0.1% trifluoroacetic acid in 2% acetonitrile. The 50-minute system suitability gradient consisted of 2 to 40% B in 30 minutes, 40 to 75% in 5 minutes, 75 to 100% B in 15 minutes, followed by a wash and a 30-minute column equilibration. The trap and column were maintained at a constant temperature of 50°C within a heated source (CorSolutions) and electrosprayed into a Thermo Orbitrap Eclipse Tribrid Mass Spectrometer with the application of a distal 2 kV spray voltage. The scan cycle included one 30,000 resolution full-scan mass spectrum (*m/z* 400–810) followed by 17 PRM MS/MS spectra targeting 2 BSA peptides and the 15 heavy labeled PRTC peptides at 15,000 resolution, AGC target of 5e4, 22 ms maximum injection time, and 30% normalized collision energy with a 2 *m/z* isolation window.

The target mass list for the parallel reaction monitoring (PRM) method included 17 entries (all z = 2): 409.7 *m/z* (ATEEQLK - light), 422.7 *m/z* (IGDYAGIK - heavy), 451.2 *m/z* (DIPVPKPK - heavy), 493.7 *m/z* (SSAAPPPPPR - heavy), 496.2 m/z (HVLTSIGEK - heavy), 498.8 *m/z* (LTILEELR - heavy), 558.3 *m/z* (GLILVGGYGTR - heavy), 573.3 *m/z* (NGFILDGFPR - heavy), 582.3 *m/z* (LVNELTEFAK - light), 586.8 *m/z* (SAAGAFGPELSR - heavy), 613.3 *m/z* (GISNEGQNASIK - heavy), 680.3 *m/z* (ELASGLSFPVGFK - heavy), 695.8 *m/z* (TASEFDSAIAQDK - heavy), 745.3 *m/z* (SFANQPLEVVYSK - heavy), 773.8 *m/z* (ELGQSGVDTYLQTK - heavy), 787.4 *m/z* (LSSEAPALFQFDLK - heavy), and 801.4 *m/z* (GILFVGSGVSGGEEGAR - heavy).

### Example 2: MS issue on Orbitrap Fusion

The PRTC/BSA system suitability standard was as described above. The tryptic yeast proteome was obtained from Promega, which was reduced, alkylated, and digested with trypsin according to the manufacturer’s instructions.

For the yeast DDA runs (n = 3 technical replicates per batch), one μg of yeast peptides were loaded, and for the PRM system suitability runs (5–11 technical replicates per batch), 3 µl of our system suitability standard was injected. Chromatography was as previously described, except that a Waters NanoAcquity UPLC was coupled to a Thermo Orbitrap Fusion.

Mobile phase buffer A was 0.1% formic acid in water and buffer B was 0.1% formic acid in 100% acetonitrile. The strong and weak needle washes were as described above. For DDA runs, the 90–95 minute gradient began with 2% B and consisted of 2 to 7.5% B in 10 minutes, 7.5 to 25% in 40 minutes, 25 to 60% B in 25 minutes, 60 to 95% B in 6 minutes, followed by column equilibration with 2% B for 14 minutes. For PRM runs, the 55–60 minute gradient began with 2% B and consisted of 2 to 35% B in 30 minutes, 35 to 60% in 11 minutes, 60 to 95% B in 6 minutes, followed by column equilibration with 2% B in 13 minutes. The trap and column were maintained at a temperature of 50°C using a heated source (CorSolutions) and electrosprayed into a Thermo Orbitrap Fusion Mass Spectrometer with the application of a distal 2 kV spray voltage.

For the DDA runs, a cycle of one 120,000 resolution MS1 scan (400–1600 *m/z*) was followed by data-dependent high energy collisional dissociation (HCD) fragmentation to acquire MS/MS spectra targeting the most intense ions. In the precursor scan, we used a charge state filter of +2 to +7, and sampled peaks were added to a dynamic exclusion list with a 20 second duration, the AGC target was 200,000, and a 20 ms maximum injection time. The MS/MS spectra (100–1000 *m/z*) acquired in the linear ion trap used an AGC target of 10,000, a 35 ms maximum injection time, and 30% normalized collision energy. For the PRM runs, a precursor scan (400–1600 *m/z*) was followed-up with a set of targeted MS/MS scans. MS/MS spectra were acquired in a linear ion trap using low energy beam CID (HCD) fragmentation, with an AGC target of 10,000, a maximum injection time of 35 s, and a 30% normalized collision energy.

We assessed the chromatography, retention times, and peak areas of 15 PRTC peptides and 2 BSA peptides in the PRM system suitability runs using Skyline (version 22.2). The raw data, Skyline document, Skyline report files, and the processed data used as input to generate Figure 3 are available on PanoramaWeb.

Using a Nextflow pipeline (https://github.com/mriffle/nf-teirex-dda, revision 068f68323c9f9a181175a81ee796ef4a3373b5ed), the DDA MS data were converted to mzML with msConvert^57^, peptides identified using Comet^58,59^, version 2023.01 rev. 2 (uwpr.github.io/Comet/releases/release_202301), q-values and posterior error probabilities at the peptide-spectrum match (PSM)level were acquired using Percolator^60^ version 3.06 (github.com/percolator/percolator/releases), and uploaded to Limelight^61^ for visualization and dissemination. Searches were performed using a Saccharomyces cerevisiae reference proteome FASTA file (Uniprot Proteome ID: UP000002311, downloaded January 27, 2024) appended with the internal control yeast enolase 1 and a common list of contaminants generated in-house. The raw data, parameter files used in Nextflow DDA analyses, and the processed data downloaded from Limelight used as input to generate Figure 3 are available on PanoramaWeb. The analyzed DDA data is freely accessible on Limelight (Project ID = 131).

### Example 3: Sample processing issue on Orbitrap Lumos Tribrid

Sample preparations of commercial human CSF (Golden West Biologicals): two different functionalized magnetic bead preparations based on in series-digestion with protein aggregation (labels: 1BD and 2BD), S-trap column digestion and clean-up (label: STR), and in-solution digestion with Rapigest SF (label: ISD), and mixed-mode solid phase extraction clean-up (label: MIX). A large pool of Golden West human CSF was aliquoted into 50 μL volumes of 8 replicates for each sample preparation type.

For the 1BD sample preparation each 50 μL aliquot of human CSF was resuspended in 8M Urea, 150 mM NaCl, 100 mM Tris pH 8.5 with 800 ng of yeast enolase protein (Sigma-Aldrich) added as an internal protein control. Processing included reduction with 10 mM dithiothreitol (DTT) (30°C, 10 minutes) and alkylation with 15 mM iodoacetamide (IAA) (25°C, 30 minutes). Proteins were aggregated on 50 μL of 10 μg/μL MagReSyn carboxylate beads (ReSyn Biosciences) with 50% ethanol, washed three times with 80% ethanol, and digested to peptides with trypsin (1:25) in 25 mM ammonium bicarbonate, using a Thermo KingFisher Flex. Samples were acidified to a final concentration of 0.1% formic acid.

For the 2BD sample preparation each 50 μL aliquot of human CSF was resuspended in 2% SDS, 100 mM Tris pH 8.5 with 800 ng of yeast enolase protein (Sigma-Aldrich) added as an internal protein control. Processing included reduction with 10 mM DTT (60°C, 10 minutes) and alkylation with 15 mM IAA (25°C, 30 minutes). Proteins were aggregated on 25 μL of 20 μg/μL MagReSyn hydroxyl beads (ReSyn Biosciences) with 70% acetonitrile, washed three times with 95% acetonitrile, washed twice with 70% ethanol, and digested to peptides with trypsin (1:25) in 50 mM ammonium bicarbonate, using a Thermo KingFisher Flex. Samples were acidified to a final concentration of 0.1% formic acid.

For the STR sample preparation each 50 μL aliquot of human CSF was resuspended in 50 μL of 2X lysis buffer of 10% SDS, 100 mM triethylammonium bicarbonate (TEAB) with 2X Halt protease and phosphatase inhibitors (Thermo Scientific) with 800 ng of yeast enolase protein (Sigma-Aldrich) added as an internal protein control. Processing included reduction with 20 mM DTT (60°C, 10 minutes) and alkylation with 40 mM IAA (25°C, 30 minutes). Lysates were prepared for S-trap column (Protifi) binding by the addition of 1.2% phosphoric acid and 350 μL of binding buffer (90% methanol, 100 mM TEAB). The acidified lysate was bound to the column incrementally. This was followed by 3 wash steps with binding buffer to remove SDS, 3 wash steps with 50:50 methanol:chloroform to remove lipids, and a final wash step with binding buffer. Trypsin (1:25) in 50mM TEAB was added to the S-trap column for digestion at 47°C for one hour. Hydrophilic peptides were eluted with 50 mM TEAB followed by hydrophobic peptides with a solution of 50% acetonitrile in 0.2% formic acid. Eluates were pooled, speed vacuumed, and resuspended in 0.1% formic acid.

For the ISD sample preparation each 50 μL aliquot of human CSF was resuspended in 0.2% RapiGest SF (Waters) in 50 mM ammonium bicarbonate 5 with 800 ng of yeast enolase protein (Sigma-Aldrich) added as an internal protein control. Processing included reduction with 10 mM DTT (60°C, 30 minutes), alkylation with 15 mM IAA (25°C, 30 minutes) and quenching with 10 mM DTT. Samples were digested in trypsin (1:25) in 50 mM ammonium bicarbonate at 37°C for 16 hours. Samples were quenched with 200 mM HCl and cleaned with mixed-mode ion exchange (MCX) columns (Waters).

One μg of each digested sample and 150 femtomole of Pierce Retention Time Calibrant (PRTC) was loaded onto a 150 µm fused silica Kasil1 (PQ Corporation) fritted microcapillary trap loaded with 3.5 cm of 3 µm Reprosil-Pur C18 (Dr. Maisch) reverse-phase resin coupled with an 75 µm inner diameter picofrit (New Objective) analytical column containing 30 cm of 3 µm Reprosil-Pur C18 attached to a Thermo EASY-nLC 1200. The PRTC was also used to assess the quality of the column before and during analysis. We analyzed four of these system suitability runs prior to any sample analysis. After every six to eight sample runs, another system suitability run was analyzed. Buffer A is 0.1% formic acid in water and buffer B is 0.1% formic acid in 80% acetonitrile. The strong needle wash was 50% acetonitrile, and the weak needle was 0.1% trifluoroacetic acid in 2% acetonitrile. The 40-minute system suitability gradient consisted of 0 to 16% B in 5 minutes, 16 to 35% in 20 minutes, 35 to 75% B in 5 minutes, 75 to 100% B in 5 minutes, followed by a wash of 9 minutes and a 30-minute column equilibration. The 110-minute sample LC gradient consists of a 2 to 7% for 1 minute, 7 to 14% B in 35 minutes, 14 to 40% B in 55 minutes, 40 to 60% B in 5 minutes, 60 to 98% B in 5 minutes, followed by a 9-minute wash and a 30-minute column equilibration. Peptides were eluted from the column with a 50°C heated source (CorSolutions) and electrosprayed into a Thermo Orbitrap Fusion Lumos Mass Spectrometer with the application of a distal 3 kV spray voltage. For the system suitability analysis, a cycle of one 120,000 resolution full-scan mass spectrum (350–2000 *m/z*) followed by a data-independent MS/MS spectra on the loop count of 76 data-independent MS/MS spectra using an inclusion list at 15,000 resolution, AGC target of 4e5, 20 millisecond (ms) maximum injection time, 33% normalized collision energy with an 8 *m/z* isolation window. For the sample digest, data were collected using data-independent acquisition (DIA) strategies. First, a chromatogram library of 6 independent injections was analyzed from a pool of all samples within a batch. For each injection, a cycle of one 120,000 resolution full-scan mass spectrum with a mass range of 110 *m/z* (395–505 *m/z*, 495–605 *m/z*, 595–705 *m/z*, 695–805 *m/z*, 795–905 *m/z*, or 895–1005 *m/z*) followed by a data-independent MS/MS spectra on the loop count of 26 at 30,000 resolution, AGC target of 4e5, 60 ms maximum injection time, 33% normalized collision energy with a 4 *m/z* overlapping isolation window. The chromatogram library data was used to quantify proteins from individual sample runs. These individual runs consisted of a cycle of one 120,000 resolution full-scan mass spectrum with a mass range of 350–2000 *m/z*, AGC target of 4e5, 100 ms maximum injection time followed by a data-independent MS/MS spectra on the loop count of 76 at 15,000 resolution, AGC target of 4e5, 20 ms maximum injection time, 33% normalized collision energy with an overlapping 8 *m/z* isolation window. Application of the mass spectrometer and LC solvent gradients were controlled by the ThermoFisher Xcalibur data system.

System suitability and internal controls in samples and external control samples were imported into Skyline (version 22.2) using similar settings for PRM and DIA data.

### Example 4: LC issue on Orbitrap Eclipse Tribrid

Human plasma was resuspended in 2% SDS, 50 mM Tris pH 8.5 with 1X Halt protease and phosphatase inhibitors (Thermo Scientific). Protein concentration was measured using the Pierce BCA assay (Thermo Scientific). Samples were prepared with 50 μg of homogenate and 800 ng of yeast enolase protein (Sigma) as an internal protein control. Processing included reduction with 10 mM DTT (60°C, 10 minutes) and alkylation with 15 mM IAA (25°C, 30 minutes). Proteins were aggregated on MagReSyn Hydroxyl beads (ReSyn Biosciences), washed, and digested to peptides, in a 96-well plate using a Thermo KingFisher Flex. Samples were acidified to a final concentration of 0.1% formic acid.

One μg of each digested sample and 150 femtomole of Pierce Retention Time Calibrant (PRTC) was loaded onto a 150 µm fused silica Kasil1 (PQ Corporation) fritted microcapillary trap loaded with 3.5 cm of 3 µm Reprosil-Pur C18 (Dr. Maisch) reverse-phase resin coupled with an 75 µm inner diameter picofrit (New Objective) analytical column containing 30 cm of 3 µm Reprosil-Pur C18 attached to a Thermo EASY-nLC 1200. The PRTC was also used to assess the quality of the column before and during analysis. We analyzed four of these system suitability runs prior to any sample analysis. After every eight sample runs, another system suitability run was analyzed. Buffer A is 0.1% formic acid in water and buffer B is 0.1% formic acid in 80% acetonitrile. The strong needle wash was 50% acetonitrile, and the weak needle was 0.1% trifluoroacetic acid in 2% acetonitrile. The 50-minute system suitability gradient consisted of 2 to 40% B in 30 minutes, 40 to 75% in 5 minutes, 75 to 100% B in 15 minutes, followed by a wash and a 30-minute column equilibration. The 100-minute sample LC gradient consists of a 2 to 40% for 80 minutes, 40 to 75% B in 10 minutes, 75 to 100% B in 10 minutes, followed by a 30-minute wash and column equilibration. Peptides were eluted from the column with a 50°C heated source (CorSolutions) and electrosprayed into a Thermo Eclipse Tribrid Mass Spectrometer with the application of a distal 3 kV spray voltage. For the system suitability analysis, a cycle of one 30,000 resolution full-scan mass spectrum (400–810 *m/z*) followed by a data-independent MS/MS spectra on the loop count of 20 data-independent MS/MS spectra using an inclusion list at 15,000 resolution, AGC target of 5e4, 22 ms maximum injection time, 30% normalized collision energy with a 2 *m/z* isolation window with a mass list table for the 2 BSA peptides and the 15 heavy labeled PRTC peptides. For the sample digest, first a chromatogram library of 6 independent injections was analyzed from a pool of all samples within a batch. For each injection, a cycle of one 30,000 resolution full-scan mass spectrum with a mass range of 110 *m/z* (395–505 *m/z*, 495–605 *m/z*, 595–705 *m/z*, 695–805 *m/z*, 795–905 *m/z*, or 895–1005 *m/z*) followed by a data-independent MS/MS spectra on the loop count of 25 at 30,000 resolution, AGC target of 5e5, 54 ms maximum injection time, 27% normalized collision energy with a 4 *m/z* overlapping isolation window. The chromatogram library data was used to quantify proteins from individual sample runs. These individual runs consisted of a cycle of one 30,000 resolution full-scan mass spectrum with a mass range of 350–1005 *m/z*, AGC target of 4e5, 50 ms maximum injection time followed by a data-independent MS/MS spectra on the loop count of 75 at 15,000 resolution, AGC target of 4e5, 22 ms maximum injection time, 27% normalized collision energy with an overlapping 8 *m/z* isolation window. Application of the mass spectrometer and LC solvent gradients were controlled by the ThermoFisher Xcalibur data system.

System suitability and internal controls in samples and external control samples were imported into Skyline (version 22.2) using similar settings for PRM and DIA data.

### Example 5: Combining system suitability and internal quality controls on Orbitrap Lumos Tribrid

Mouse brain micropunches were homogenized in 30 μL of 5% SDS, 50 mM triethylammonium bicarbonate (TEAB) with 1X Halt protease and phosphatase inhibitors (Thermo Scientific) in a Barocycler 2320 EXT (Pressure Biosciences Inc.) for 30 repeat cycles (20 sec at 45k psi, 10 sec at ambient pressure) at 35°C. Protein concentration was measured using the Pierce BCA assay (Thermo Scientific). Samples were prepared with 25 μg of homogenate and 400 ng of yeast enolase protein (Sigma) as an internal protein control. Processing included reduction with 20 mM DTT (60°C, 10 minutes) and alkylation with 40 mM IAA (25°C, 30 minutes). Lysates were prepared for S-trap column (Protifi) binding by the addition of 1.2% phosphoric acid and 350 μL of binding buffer (90% methanol, 100 mM TEAB). The acidified lysate was bound to the column incrementally. This was followed by 3 wash steps with binding buffer to remove SDS, 3 wash steps with 50:50 methanol:chloroform to remove lipids, and a final wash step with binding buffer. Trypsin (1:10) in 50mM TEAB was added to the S-trap column for digestion at 47°C for one hour. Hydrophilic peptides were eluted with 50 mM TEAB followed by hydrophobic peptides with a solution of 50% acetonitrile in 0.2% formic acid. Eluates were pooled, speed vacuumed, and resuspended in 0.1% formic acid.

One μg of each digested sample and 150 femtomole of Pierce Retention Time Calibrant (PRTC) was loaded onto a 150 µm fused silica Kasil1 (PQ Corporation) fritted microcapillary trap loaded with 3.5 cm of 3 µm Reprosil-Pur C18 (Dr. Maisch) reverse-phase resin coupled with an 75 µm inner diameter picofrit (New Objective) analytical column containing 30 cm of 3 µm Reprosil-Pur C18 attached to a Thermo EASY-nLC 1200. The PRTC was also used to assess the quality of the column before and during analysis. We analyzed four of these system suitability runs prior to any sample analysis. After every six to eight sample runs, another system suitability run was analyzed. Buffer A is 0.1% formic acid in water and buffer B is 0.1% formic acid in 80% acetonitrile. The strong needle wash was 50% acetonitrile, and the weak needle was 0.1% trifluoroacetic acid in 2% acetonitrile. The 40-minute system suitability gradient consisted of 0 to 16% B in 5 minutes, 16 to 35% in 20 minutes, 35 to 75% B in 5 minutes, 75 to 100% B in 5 minutes, followed by a wash of 9 minutes and a 30-minute column equilibration. The 110-minute sample LC gradient consists of a 2 to 7% for 1 minute, 7 to 14% B in 35 minutes, 14 to 40% B in 55 minutes, 40 to 60% B in 5 minutes, 60 to 98% B in 5 minutes, followed by a 9-minute wash and a 30-minute column equilibration. Peptides were eluted from the column with a 50°C heated source (CorSolutions) and electrosprayed into a Thermo Orbitrap Fusion Lumos Mass Spectrometer with the application of a distal 3 kV spray voltage. For the system suitability analysis, a cycle of one 120,000 resolution full-scan mass spectrum (350–2000 *m/z*) followed by a data-independent MS/MS spectra on the loop count of 76 data-independent MS/MS spectra using an inclusion list at 15,000 resolution, AGC target of 4e5, 20 millisecond (ms) maximum injection time, 33% normalized collision energy with an 8 *m/z* isolation window. For the sample digest, first a chromatogram library of 6 independent injections was analyzed from a pool of all samples within a batch. For each injection, a cycle of one 120,000 resolution full-scan mass spectrum with a mass range of 110 *m/z* (395–505 *m/z*, 495–605 *m/z*, 595–705 *m/z*, 695–805 *m/z*, 795–905 *m/z*, or 895–1005 *m/z*) followed by a data-independent MS/MS spectra on the loop count of 26 at 30,000 resolution, AGC target of 4e5, 60 ms maximum injection time, 33% normalized collision energy with a 4 *m/z* overlapping isolation window. The chromatogram library data was used to quantify proteins from individual sample runs. These individual runs consisted of a cycle of one 120,000 resolution full-scan mass spectrum with a mass range of 350–2000 *m/z*, AGC target of 4e5, 100 ms maximum injection time followed by a data-independent MS/MS spectra on the loop count of 76 at 15,000 resolution, AGC target of 4e5, 20 ms maximum injection time, 33% normalized collision energy with an overlapping 8 *m/z* isolation window. Application of the mass spectrometer and LC solvent gradients were controlled by the ThermoFisher Xcalibur data system.

System suitability and internal controls in samples and external control samples were imported into Skyline (version 22.2) using similar settings for PRM and DIA data.

### Example 6: Assessing quantitative results on Orbitrap Eclipse Tribrid with external quality controls

Lumbar CSF from 280 patients were divided into four major groups: 1) Healthy Control, 2) Alzheimer’s disease/mild cognitive impairment, 3) Parkinson’s disease cognitively normal and 4) Parkinson’s disease cognitively impaired. Each row of half a 96-well plate contained 10 balanced and randomized CSF samples and two external QCs. One external inter-batch control was a pool of 50 patients representing all 4 groups and the other external inter-experiment QC was a commercially available pool of CSF. These controls were processed with the samples and used to evaluate the technical precision within and between each batch prior to and following normalization and batch adjustment.

The CSF samples (25 µg) were resuspended in SDS lysis buffer (working concentration of 1% SDS, 50 mM Tris, pH 8.5) with 400 ng of yeast enolase as a protein internal control to assess sample digestion. Samples and external controls were reduced with 40 mM DTT (10 minutes at 60 °C), alkylated with 80 mM iodoacetamide (30 minutes at room temperature), and quenched with 40 mM DTT. Proteins were aggregated on MagReSyn Hydroxyl beads by diluting samples to 70% acetonitrile, washed three times with 95% acetonitrile, washed twice with 70% ethanol, and digested to peptides with 1:10 trypsin at 47°C for one hour using a Thermo KingFisher Flex. Samples were spiked with 150 fmol of PRTC per injection volume. Peptides were separated using reverse-phase chromatography with a Thermo Easy nano-LC and electrosprayed into a Thermo Orbitrap Eclipse Tribrid analyzed using a 12 *m/z* staggered DIA isolation scheme. The data were demultiplexed to 6 *m/z* with ProteoWizard with settings of “overlap_only” and Mass Error = 10.0 ppm. A Prosit library is generated using the Uniprot human canonical FASTA (Proteome ID: UP000005640) and NCE setting=33^62,63^. Using EncyclopeDIA (version 2.12.30), the 6x GPF-DIA acquisitions from each plate batch are combined to a single global GPF library, shifts in retention times are aligned, and batch-specific GPF libraries are generated. The Prosit library is empirically corrected by searching the batch-specific GPF libraries as described by Searle et al^64,65^. Default EncyclopeDIA settings are used: (10 ppm tolerances, trypsin digestion, and higher-energy collisional dissociation [HCD] b- and y-ions). The results from this analysis were saved as a “Chromatogram Library’’ in EncyclopeDIA’s eLib format. The “wide window” DIA runs are analyzed using EncyclopeDIA requiring a minimum of 3 quantitative ions and filtering peptides with q-value ≤ 0.01 using Percolator 3.01. After analyzing each file individually, EncyclopeDIA is used to generate a “Quant Report” which stores all the detected peptides, integration boundaries, quantitative transitions, and statistical metrics from all runs in an eLib format.

The Quant Report eLib library was imported into Skyline using the human Uniprot FASTA as the background proteome to map peptides to proteins, perform peak integration, manual evaluation, and generate reports. The sample mzML files were then imported into Skyline. A csv file of peptide level total area fragments (TAFs) for each replicate was exported from Skyline using the custom reporting capabilities of the document grid. Peptide quantities were then median normalized, and batch corrected. All levels of data are available via PanoramaWeb. The coefficient of variation calculated for all peptides in the inter-batch QC improved 6.3% after normalization and batch adjustment at the peptide-level and 16.9% at the protein level. We also use internal process controls to assess sample preparation and monitor data collection. These controls were necessary, as the data collected encompassed seven batches analyzed on separate LC columns and traps over a three-month period.

### Data Accessibility and Figure Generation

Raw files (DIA, PRM, DDA), Skyline documents, processed results (DIA, PRM) used as input for figure generation, FASTA files, EncyclopeDIA files, and metadata are available on PanoramaWeb (panoramaweb.org/maccoss-sample-qc-system-suitability.url). The data DOI is 10.6069/hmtb-tp04. The processed DDA results in Figure 3 are freely accessible on Limelight^61^ under the identifier Project 131. The dataset are also registered through the ProteomeXchange with the unique identifier PXD051318.

**Figure 2:**
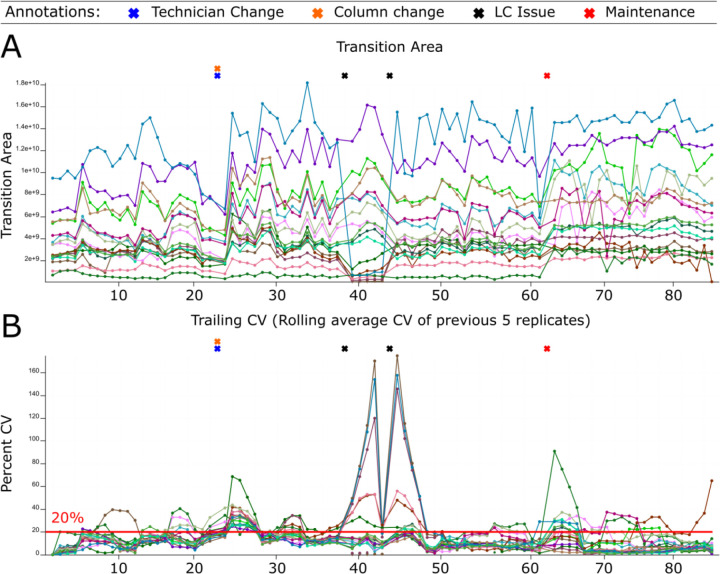
Monitoring LC-MS system performance longitudinally. System suitability tests are targeted (PRM) runs tracking the LC-MS response of consistent quantities of control peptides before, during, and after experimental runs. Raw data files are automatically uploaded and viewed online using Skyline with Panorama AutoQC. This facilitates longitudinal assessment of the system’s function via numerous metrics including precursor area, transition area, precursor/transition area ratios, retention time, mass accuracy, and more. This approach can identify functional deficits that are not always apparent in the standard approach that simply monitors DDA peptide identification counts. We show system suitability runs from an Orbitrap Eclipse Tribrid from March 1, 2022 - April 10, 2022 including the technician annotations and two experimental metrics: (A) the transition area of individual runs and (B) the trailing CV of the previous 5 replicates. A column and technician change occurred on March 13 (orange and blue “X”s). The transition area (A) and precursor area (not shown) dropped starting the morning of March 21st (first black “X”). The technician postulated there was an issue with the LC solvents, but queued up system suitability runs to confirm the system decline. The trailing CV also clearly showed the change in the transition area (B) and precursor area (not shown). After several hours when performance had not improved, the sample pump buffer was replaced (Second black “X”). Shortly after, the system stabilized. On April 1, routine maintenance to clean the quadrupole was performed and a new set of runs were started.

**Figure 3:**
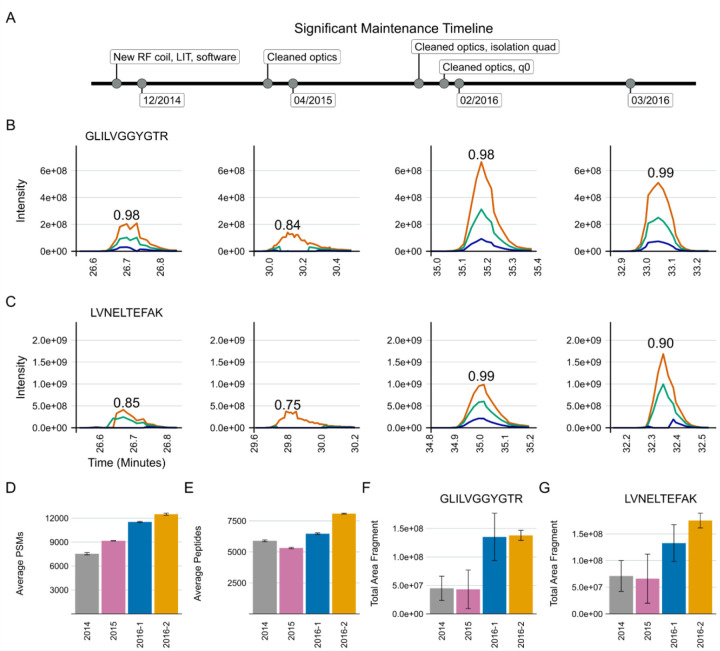
DDA spectral counting and peptide identifications fail to detect severe instrument failure. An Orbitrap Fusion exhibited inconsistent data collection for years. Typically, after cleaning various ion optics the system performance would be evaluated using DDA spectral counting and targeted PRM methods. Three different sessions spanning approximately 16 months from this time are shown to illustrate why targeted (PRM) system suitability runs were adopted. Major periods of maintenance in close proximity to these runs are shown in (A). We frequently, but inconsistently, observed the loss of ^13^C isotope peaks M+1 and M+2 precursors in the MS1 chromatograms of peptides in our PRM system suitability tests. The idotp values are printed on the chromatograms. Many peptides exhibited these issues during this time, but one representative replicate of GLILVGGYGTR (B) and LVNELTEFAK (C) are shown for brevity. Cleaning the optics in April 2015 only seemed to make the PRM chromatograms (B and C, second panels) worse. At the same time, DDA-identified PSMs increased (D) and identified peptides decreased slightly (E). The total area fragment of GLILVGGYGTR (F) and LVNELTEFAK (G) did not improve. In 2016, additional ion optics were cleaned (A). Most notable was some build up on the bent quad (q0) that was removed. At that point, the system began to improve, and we saw signal intensities improve in the 2016 batches in the DDA (D) and PRM (B-G) runs. However, it was clear that the underlying hardware issue had not been resolved as we still observed inconsistent, seemingly random loss of M+1 and M+2 signal in 2016 while the DDA-derived PSMs and peptide identifications continued to climb, suggesting these approaches do not serve to capture even significant instrumentation issues.

**Figure 4:**
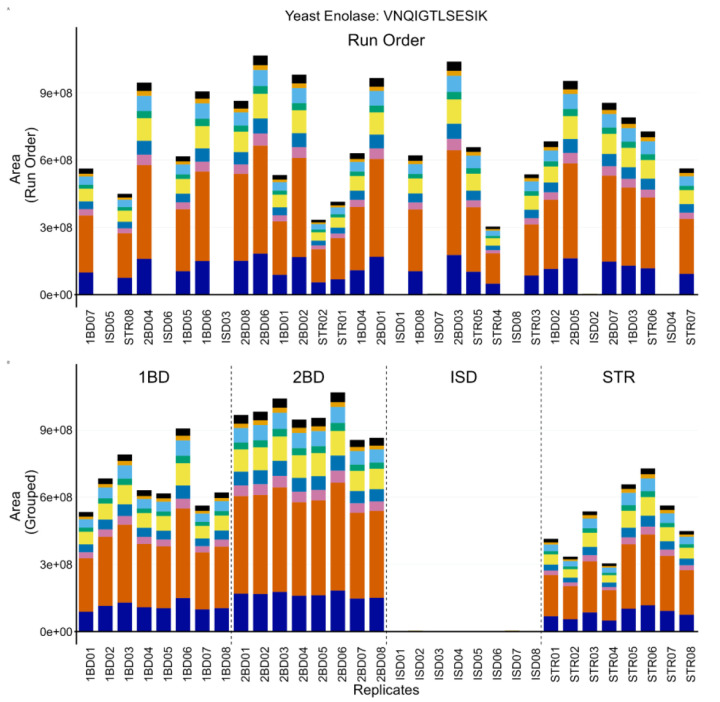
Internal QCs added to experimental samples are used to assess the entire process from lysis to LC-MS. Four different sample preparation protocols were tested - two on-bead protocols (1BD and 2BD), S-trap (STR), and in-solution (ISD). All the samples were spiked with ENO as the protein internal QC to evaluate the overall processing. The transition peak area of VNQIGTLSESIK is shown here. The run order for the 8 replicates from each condition were randomized, as shown in panel (A). At inconsistent intervals, numerous samples were missing ENO peptides, whereas the PRTC peptides looked normal (Supplementary Figure 1). The cause of the missing enolase peptides became clear when the samples were grouped by sample preparation (B), revealing that there was an issue with the ISD sample preparation method. We later found that our Rapigest stock, which was one of the only reagents different between the four protocols, was expired and could have hindered denaturation, and thus digestion, in the ISD samples.

**Figure 5:**
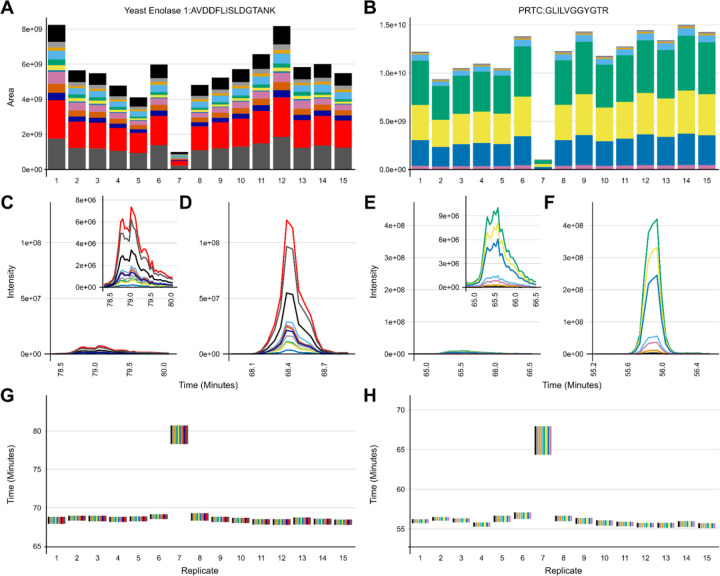
The two internal QCs can help distinguish between sample preparation versus LC-MS problems. In a series of plasma samples analyzed on an Eclipse Tribrid, one injection denoted 7 was found to have a reduced peak intensity and altered retention time across all of the internal QC peptides. One peptide from each control is shown here for brevity. In ENO, AVDDFLISLDGTANK transition areas were reduced (A), chromatography was poor (C), and the retention time was shifted (G). GLILVGGYGTR from PRTC shared a reduction in peak area (B), poor chromatography (E), and shifted retention time (H). Upon manual inspection of the LC after injection 7, the technician determined that the outlet line coming from the injection valve had partially clogged. Once the repair was completed, three system suitability injections (not shown) were found to be stable and with comparable signal intensity to before the clogged line. Another sample was injected (08) and then the previous sample was reinjected into the system and is shown as 9. The transition peak areas and chromatography of AVDDFLISLDGTANK (A, D) and GLILVGGYGTR (B, F) were normal relative to the previous samples, and the retention times were again in line with other samples (G, H).

**Figure 6:**
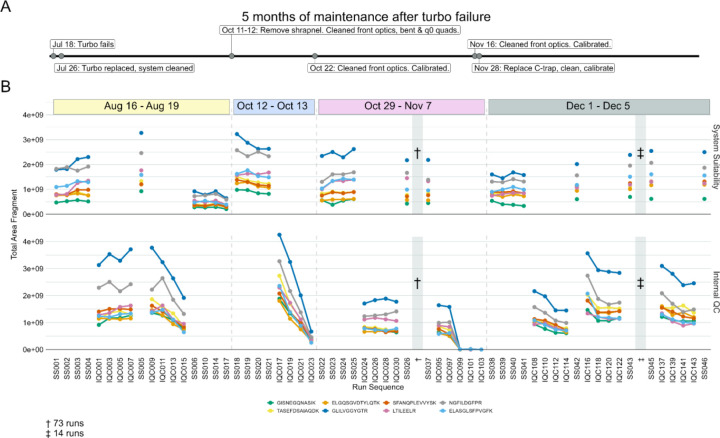
Combining system suitability injections and sample internal QCs facilitate real-time monitoring of the system. On an Orbitrap Lumos Tribrid, a significant turbo failure led to months of inconsistent and unpredictable data collection. A rough summary of the major maintenance steps during 5 months of troubleshooting post-turbo failure are shown here (A). Between August - December, signal intensity would unpredictably begin decreasing with each injection. Eight PRTC peptides from system suitability runs (B, top) and sample internal QCs (B, bottom) illustrate this in four different runs. Only a subset of all runs are shown here to improve visibility, but the trends are representative. The full dataset including all runs for PRTC, ENO, and BSA are available on Panorama. In August, after 7 sample injections (IQC001-IQC007) and 5 system suitability injections (SS001-SS005) the system was found to be stable. As additional samples were run (IQC009-IQC015), the signal intensity dropped significantly. This reduced performance was confirmed with system suitability injections (SS006-SS017). The system was taken offline. Additional metal debris from the turbo failure that was missed in earlier maintenance was removed, and the front optics and quads were cleaned. After calibration, the same issue of rapidly declining signal was observed in mid-October (SS018-IQC023). After taking the system down and cleaning the optics again, the system seemed stable for 75 runs (SS026-SS037) until the signal intensity declined rapidly while running samples IQC097-IQC103. The system was taken down again. In late November metal debris was found lodged in the C-trap, and it was replaced along with another thorough cleaning. We then observed a return to expected signal stability and intensity (SS038 through SS046) relative to matched sample batches run prior to the system failing.

**Figure 7:**
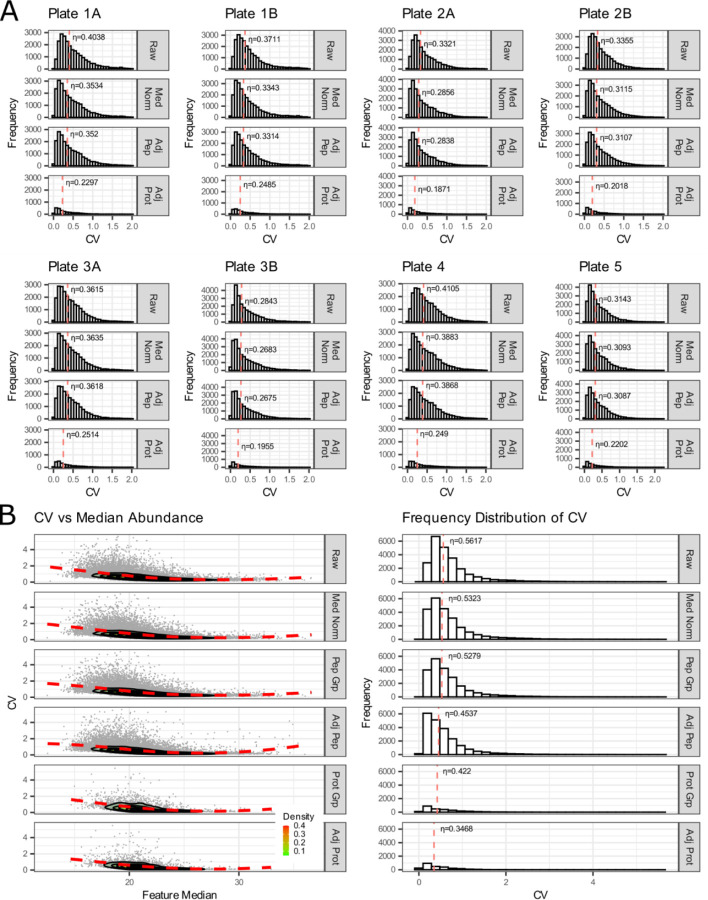
Assessment of quantitative results using median normalization and batch correction of inter-batch external QCs. (Top panel or 7A) The effect of median normalization and batch correction of peptide and protein coefficient of variance (CV) from all the pooled lumbar CSF inter-batch external QCs within each plate (batch). The median coefficient of variation (η) is indicated by the dashed red line. (Bottom panel or 7B) The effect of median normalization and batch correction of peptide and protein coefficient of variance from all the pooled lumbar CSF inter-batch external QCs from all plates. The relationship between coefficient of variation and the log2 median abundance is visualized with a LOESS fit of a contoured density plot (red line).

The Inkscape files and Panorama AutoQC images used to generate Figures 1 and 2 are available on Panorama and GitHub. Inkscape was used for minor aesthetic changes, panel labeling, and resizing of figures. Input files for R scripts >25 MB are available in individual figure folders on PanoramaWeb. The input files <25 MB and the R code to generate Figures 3–6 (maccoss_qc_figures_3_4_5_6_S1.Rmd) and Figure 7 (plot_7a.r, plot_7b.r) are freely available for download on GitHub: (github.com/uw-maccosslab/maccoss-sample-qc-system-suitability/).

## Results

### Experimental Design

Before any sample preparation can begin, numerous QC steps should be implemented. This is especially important anytime multiple sample groups are being considered. First, internal protein and peptide QCs should be tested in the sample matrix of interest to ensure optimum performance. The composition of pooled external QC samples are planned and the pools generated ahead of time. More information about the generation of these external QC samples is described below in Figure 7. Next, all experimental samples and external QCs are batched randomly and balanced based on the experimental covariates. As an example in biological studies, we ensure that samples are spaced within a batch or on a plate such that individuals of different sex, age, and treatment or condition group are well-spaced on a plate and not processed in close proximity to one another. The order in which samples are processed within plates or batches is also maintained as the run order on the instrument rather than randomizing the samples a second time. This can help reduce the contribution of variation associated with batch effects and run order. As part of this process, two external QC samples are included in each row, but they are not placed within the same column on a plate. Other groups have reviewed best practices for sample batching and block randomization^43,44^ in proteomics.

### System Suitability

Ensuring optimal system function prior to data collection and assessment is paramount. We have found that the combined use of system suitability injections and the inclusion of internal QCs in experimental samples are useful to monitor the function of the LC-MS/MS during an experiment. Importantly - these two controls used in parallel help technicians determine if changes to the system need to be made.

The system suitability control serves three functions: evaluation of the system at the start of an experiment, near real-time assessment during an experiment, and the longitudinal tracking of the system. Historically, our system suitability runs most often used shorter LC gradients coupled to PRM MS runs composed of known quantities of bovine serum albumin (BSA) and Pierce^™^ Peptide Retention Time Calibration Mixture (PRTC) peptides. We automatically upload system suitability data to shared online databases on PanoramaWeb^56^ using Skyline^54,55^ and the Panorama AutoQC loader^12^. This process allows easy, automated, and rapidly visualized long-term tracking of numerous QC metrics across all our LC-MS systems. Levey-Jennings plots^66^ are useful to monitor individual points, while cumulative sum charts, moving range, trailing average, and trailing CV plots can capture longer-term trends in the data. A subset of tracked metrics we focus on most often include transition or precursor areas and their ratio, mass accuracy, retention time, and isotope dot product (idotp) values. Here we show Example 1 where the transition area and rolling CV during a typical month of system suitability runs on our Orbitrap Eclipse are shown in Figure 2A and 2B. Finally, we pair these data deposition and tracking with user-provided annotations and logbooks. Anytime a new technician runs an instrument, they can easily access historical data about their system to troubleshoot issues and assess how the current system performance compares to historical metrics.

In developing our approach to assessing system suitability, we have shifted to using targeted (PRM) methods that track known peptides in a reasonably simple mixture. This provides a more accurate picture of the current state of a system’s function than classical approaches which consider identifications of peptides or PSMs. In Example 2, our group had a Thermo Orbitrap Fusion coupled to a Waters NanoAcquity ultra-performance liquid chromatography (UPLC) system that exhibited inconsistency in data collection due to unknown causes from late 2014 into 2016 (Figure 3). At that time, we were using both DDA and targeted assays to assess system suitability. Historically, we and other groups would consider peptide identifications and PSMs in a commercially available or in-house complex digest measured by DDA as a fast proxy for the functioning of our MS platforms. We have since pivoted to rapid evaluation of multiple metrics of stability including the chromatography, retention time, mass accuracy, and peak intensity of a reasonably simple pooled sample generated in-house. This allows the group to evaluate differences between instruments, discern LC versus MS issues, and identify deeper problems in the MS that may not be captured by relying on identifications.

A standardized yeast proteome digest was measured with DDA across three months - December 2014, April 2015, February 2016, and March 2016. Within the same instrument runs, we measured the mixture we currently use for system suitability with PRM (600 fmol BSA, 150 fmol PRTC per injection). The targeted Skyline document and the raw files for the DDA and PRM runs are available on PanoramaWeb. The analyzed DDA results are available on Limelight. A timeline of significant maintenance on the MS during this time is provided (Figure 3A). Representative examples of PRM precursor chromatograms are shown for GISNEGQNASIK (PRTC, Figure 3B) and for LVNELTEFAK (BSA, Figure 3C) in each dataset. We frequently, but not predictably, observed a loss of M+1 and M+2 ^13^C isotope peaks in precursor chromatograms in system suitability runs beginning in 2014 (Figure 3B and 3C, left panel). This was reflected in idotp values (shown above chromatographic peaks). By 2015, after cleaning the front optics, the performance declined even further (Figure 3B and 3C, second panels). The total area fragment intensities of GLILVGGYGTR (F) and LVNELTEFAK (G) did not increase substantially. However, during this transition, the number of PSMs increased despite the worse instrument function. Peptide identifications by DDA did nominally decline, although the decrease was not commensurate with the degree of instrumentation failure. Later in 2016, additional ion optics were cleaned (A) including visible build-up on the bent quad (q0) which was removed. Signal intensities improved markedly in terms of both the DDA (D) and PRM (B-G) runs. However, it was evident that additional hardware issues were present since we still continued to observe sporadic loss of M+1 and M+2 signal in the two 2016 batches shown here. Concurrently, the PSMs and peptide identifications in the DDA runs continued to increase despite clear instrumentation issues.

### Internal quality controls to assess processing

Internal QCs are added to all experimental samples to assess protein digestion, peptide recovery, and to distinguish between sample preparation issues and post-digestion measurement problems. We most often use yeast enolase 1 (ENO) protein as a protein internal QC to monitor digestion and peptide recovery when working with mammalian samples. PRTC is spiked into samples after digestion and clean-up just before injection as a peptide internal QC.

In Example 3, we used protein and peptide internal QCs to troubleshoot while standardizing a digestion and clean-up protocol in human cerebrospinal fluid (CSF). We incorporated the same protein (ENO) and peptide (PRTC) internal QCs in different preparations of pooled CSF to enable more direct comparison between the methods tested. Four approaches were examined: two different functionalized magnetic bead preparations based on in series-digestion with protein aggregation (labels: 1BD and 2BD), S-trap digestion and clean-up (label: STR), and in-solution digestion with Rapigest SF (label: ISD) with MCX clean-up. The digestions were performed using the same pool of human CSF and run on a Thermo Orbitrap Fusion Lumos Mass Spectrometer coupled to a Thermo Easy-nLC with nanoflow chromatography.

Throughout the course of running these samples, the ISD condition had significantly reduced ENO peptide abundance. However, the PRTC peptides were consistent with the other protocols. The system suitability injections interspersed between sample runs showed no significant changes (not shown), which suggested the problem was protocol-specific rather than a problem with the LC-MS/MS system. The technician initially postulated that they had made a mistake in pipetting a crucial reagent during the ISD preparation. To confirm the performance of the four methods without ambiguity, the entire experiment was repeated with additional caution and 8 replicates of pooled CSF. The same results were observed as in the first replicate experiment described above, and a representative example is shown for the ENO peptide VNQIGTLSESIK in Figure 4. Supplemental Figure 1 includes an additional ENO peptide (AADALLLK, A and C) and two PRTC peptides (NGFILDGFPR, E and G; TASEFDSAIAQDK, F and H). Despite the abundance of caution taken by the technician, a number of runs were found to be missing appreciable levels of ENO peptides (Figure 4A and Supplemental Figure 1, A and B), but contained expected levels of PRTC (Supplemental Figure 1, E and F). When the data were re-ordered by sample group, it became clear that the ENO peptides in the ISD prep were consistently absent (Figure 4B and Supplemental Figure 1, C and D) and PRTC peptides were congruent with the other 3 protocols (Supplemental Figure 1, G and H). No significant deviations were seen in the system suitability injections throughout the run (not shown). Many of the reagents between the different preparations were shared including dithiothreitol, iodoacetamide, trypsin, ENO, and PRTC. We postulated the only reagent used exclusively in the ISD preparation was likely the source of the problem. Upon further inspection, the stock solution of 0.2% Rapigest SF (Waters) had expired. We postulated that an incomplete denaturation could hinder digestion and explain the lack of ENO peptides while PRTC peptides, which were added after digestion and clean-up, would perform as expected.

### Higher resolution measurement of system function using internal quality controls

We have found that internal QCs in our sample injections can also serve as a measure of system functionality. While our system suitability injections are frequently performed every 8 to 12 hours, depending on the technician and the experiment in question, the consistency of protein and peptide internal QC peptides can be examined after every sample injection to monitor issues as they arise.

Spontaneous issues with sample injections and the samples themselves happen. We share one such case in Example 4 from a Thermo Easy-nLC with nanoflow chromatography coupled to a Thermo Orbitrap Eclipse Mass Spectrometer. While running a series of human plasma samples, we identified a bad injection using the protein (ENO) and peptide (PRTC) internal QCs present in the samples. The problematic injection is denoted “7” in Figure 5. The peak areas of ENO (Figure 5A) and PRTC (Figure 5B) were reduced relative to the other samples, and the peak shape was markedly worse (Figure 5C and E).

Additionally, their retention times were shifted later by several minutes (Figure 5G and H). Right after sample 7 was run, manual inspection of the LC lines by the technician revealed a clogged outlet line coming from the sample injection valve. Once this was replaced, sample 7 was reinjected into the system and is denoted as sample 9. The peak areas (Figure 5A and B), peak shape (Figure 5D and F), and retention times (Figure 5G and H) in ENO and PRTC peptides were now consistent with the previous sample runs.

### Combining internal quality controls and system suitability

One might question whether it is worth the time collecting the system suitability data when the same PRTC peptides are spiked into each sample as an internal QC. However, we would argue that it is important to periodically evaluate instrument performance without the complications of varying sample matrices, sample preparation methods, or technicians. The combined use of system suitability injections and samples containing protein and peptide internal QCs allowed the technician to identify a significant system issue in Example 5.

Suspected charging was observed on an Thermo Orbitrap Fusion Lumos Mass Spectrometer coupled to a Thermo Easy-nLC 1200 with nanoflow chromatography. We spent approximately 5 months troubleshooting inconsistent system performance after a turbo pump exploded while the MS was running under vacuum, which dispersed debris into the system. Key maintenance that occurred during this time are shown in Figure 6A in relation to a series of system suitability and sample runs from four instrument runs spanning about 4 months. The sample injections were all mouse brain peptides acquired using DIA in 110-minute gradient runs. Eight PRTC peptides are shown in Figure 6B. The runs are broken down by system suitability runs (SS, top), and internal QCs from sample runs (IQC, bottom). To simplify the figure, only a subset of all the runs from August-December are shown. The runs are representative of observed trends in the full dataset, which is available on PanoramaWeb for PRTC, ENO, and BSA peptides.

After the turbo pump failed, the MS was stopped, and the internal components cleaned thoroughly. After passing vendor calibration and other in-house assessments, the experimental runs were restarted. Although the system was initially consistent (SS001-SS005 and IQC001-IQC007), the peak areas of PRTC and ENO (not shown) in sample injections started to decrease rapidly (IQC009-IQC015) with each injection. The subsequent system suitability injections (SS06-SS17) queued by the technician confirmed a loss of system function relative to baseline. The system was taken offline (first dashed line). More metal debris was found and removed, and the front optics, bent quad, and q_0_ were cleaned. After this maintenance and recalibration, the system suitability runs were consistent at the start of this second run (SS018-SS021), but PRTC and ENO (not shown) declined quickly during the first set of sample runs (IQC017-IQC023). The system was taken offline again, and additional maintenance was performed including further searches for debris and cleaning the optics followed by re-calibration (second dashed line). The same issue occurred near the end of a longer run (SS022-IQC103) where 73 intermediate, stable runs are denoted by “†”. The final sample injections exhibited a loss in sensitivity. The run was halted, and the system was taken offline. Upon closer inspection of the instrument interior, additional metal debris was found lodged inside of the C-trap, and it was replaced (third dashed line). The system was stably restored to normal function. The same samples which had declining sensitivity from August 16 through November 7 were reinjected and did not exhibit the same behaviors here. The system suitability and samples ran in December (SS038-SS046) reflected historical performance for that instrument in terms of the system suitability runs and the other sample batches from the study.

### Assessing Quantitative Results

Once the process and system are verified to have functioned as expected, we assess our quantitative results using a combination of internal and external QCs. An inter-batch external QC is an additional sample type of the same or similar matrix that is processed to evaluate sample preparation and variability within a batch. The inter-experiment external QC can assess CV across experiments and is processed in parallel with the samples and inter-batch external QC. The internal protein control in these external QCs can be used to evaluate how consistent sample preparation was within a batch and across batches (not shown). We share a final example, Example 6, where these controls were prepared, integrated, and applied in a human CSF study. The inter-batch external QC was created by pooling 50 human CSF patient samples from Healthy Controls, Alzheimer’s disease/mild cognitive impairment, Parkinson’s disease cognitively normal, and Parkinson’s disease cognitively impaired. The inter-experiment external QC was generated using a commercially acquired CSF stock. Both external QCs were included to assess the interplate and intraplate CVs after median normalization and batch adjustment.

We used the inter-batch external QCs to evaluate the variation and impact of normalization within and between 8 sample batches (Figure 7A). These data are examined in series beginning with level 1 (un-normalized raw peptides), level 2 (log_2_ transformed and median-normalized peptides), and level 3 (normalized and batch-corrected data) at the peptide and protein level. We are able to clearly show that the batches were broadly similar through our entire analytical pipeline and that overall variability was reduced with normalization. The controls were also used to examine the effect of normalization and batch correction on peptide and protein coefficient of variance in two ways. First, by examining the relationship between coefficient of variation and the log2 median abundance (Figure 7B, left panel) with a LOESS fit of a contoured density plot (red line), and second by examining the median coefficient of variation (η, denoted with red dashed line) of relative to median abundance (Figure 7B, right panel). The raw unnormalized data, the log2 transformed and median-normalized data, and batch corrected data are available on Panorama and GitHub.

## Discussion

Here we demonstrate real-world examples of a QC framework that incorporates long-term monitoring of LC-MS systems, the use of QCs to identify issues in sample processing and system function, and how designing experiments with integrated internal and external QCs can improve confidence in quantitative analyses. While we have used this QC framework in various stages for years, the series of case studies presented here represents the first detailed summary of the workflow in its entirety. Although we initially used DDA and spectral counting for system suitability measurements, we quickly moved on to targeted methods^67^, added internal QCs^68^, and incorporated external QCs alongside internal QCs and system suitability^69^. Additional examples of studies where this QC workflow was applied are listed on Panorama under the “Reference Information” folder. This QC framework can quickly and effectively identify issues in studies of basic biology, clinical research, and in large-scale production work. All data and analyses described in this work are available on PanoramaWeb or GitHub.

Our system suitability approach combines a reasonably simple, stable, and well-characterized reference sample, targeted MS runs before, during, and after experimental samples, and near real-time evaluation. These runs have been standardized across LC-MS instrument platforms and are longitudinally tracked with cloud-based upload and backup facilitated by PanoramaWeb and AutoQC Loader alongside detailed annotations. Using Skyline, Panorama, and AutoQC Loader, a technician can track numerous metrics including but not limited to precursor and/or transition peak areas and ratios, retention times, isotope dot products, and mass accuracy. If the system suitability runs show that both the LC and MS are functioning consistently based on the aforementioned metrics - and historical data, when available - the technician can begin running experimental samples. The initial system suitability runs serve as a baseline to monitor the system throughout a run and help identify issues more quickly as they arise. We showed in Figure 2, 3, 5, and 6 specific examples where system suitability runs were used to aid technicians in identifying problems with the LC system (Figure 2 and 5) and with the MS (Figure 3 and 6).

We settled on the use of targeted runs to assess system suitability due in large part to the rapid and straightforward analysis of targeted data in Skyline. The PRM and DDA data shown in Figure 3 provided additional confidence in relying on a targeted approach to system suitability. An Orbitrap Fusion exhibited frustratingly inconsistent loss of M+1 and M+2 precursor ions that took years to resolve. However, we found that the use of PSM number or peptide identifications in DDA runs or solely precursor and fragment ion intensities was not adequate to describe the severity of the instrumentation failure that was occurring. PSMs identified in DDA runs, in particular, were decoupled from system performance as they increased even when the system was at its worst in 2015 (Figure 3D). Metrics including PSMs and peptide identifications derived from DDA are also subject to changes throughout the data analysis pipeline. This can include inadvertent changes in the database search software or the search parameters. This would make longitudinal system suitability assessment particularly challenging. We propose that the use of PSMs or peptide identifications cannot always capture even significant instrumentation failures, and thus should not be relied upon to assess system suitability alone. Only through a more thorough analysis of the system suitability by evaluating chromatograms, precursor and fragment intensities, mass error, and retention time stability were we able to clearly identify that the issue was still present intermittently.

Protein and peptide internal QCs were highlighted in Figures 4, 5, and 6. When selecting internal QCs for a workflow, we strongly recommend assessing peptide stability in an autosampler, and peptide chromatographic consistency and signal intensity. For protein internal QCs, we also suggest evaluating digestion reproducibility. Internal QC peptides are intended for broad, lab-wise use and rigorously testing the reproducibility and stability of potential peptides in multiple sample matrices is strongly recommended. Optimization experiments to find an ideal concentration that reliably reflects instrument performance and balances cost burden is advised. Additionally, we suggest evaluating the performance of internal QCs when moving between lots to minimize ambiguity in performance assessment. The examples we highlighted here show how internal protein and peptide QCs can be used to differentiate between failed sample preparation (Figure 4), LC issues (Figure 5) or MS issues (Figure 6). Including both internal QCs in all experimental samples can be used to differentiate between a single bad injection, instrumentation failures, and a systemic problem in sample preparation and give more confidence in the results of an experiment.

Protein internal QCs are added to experimental samples early in the sample preparation protocol to examine the consistency and efficacy of sample and data processing. Heavy-labeled proteins or proteins from distant species are suitable after testing their digestion efficiency, chromatographic stability, and sequence specificity in the matrix of interest. In earlier studies, we used labeled Apolipoprotein B as a protein internal QC as it could be quantified consistently^70^. We found through internal testing that yeast enolase 1 (ENO) digested and chromatographed consistently, the peptide sequences were distinct from human enolase, and it was less costly to use at scale in our high-throughput work. We now routinely use ENO as a protein internal QC and PRTC as a peptide internal QC. However, if the experiment involves yeast or a closely-related organism, the enolase peptide signal would suffer from interference of endogenous peptides with conserved sequence and thus nullify its use as a QC. Although not discussed here, exogenous protein and peptides can also be used to monitor post-translational modification (PTM) enrichment. We have used bovine beta-casein protein coupled with ENO as a first step to distinguish between digestion deficiencies and issues with phosphopeptide enrichment. Additionally, the inclusion of heavy and light-labeled phosphopeptides spiked into a sample and described elsewhere^71,72^ can provide an estimate of the phosphopeptide enrichment efficiency.

Peptide internal QCs are added to all samples in equivalent quantities in addition to the protein internal QCs. We prefer to use stable, standard peptides that are known to perform well on our systems. Historically that has been a commercially available heavy peptide mixture (PRTC), but this could also be synthetic peptides without sequence overlap to the analytes of interest^73^. The quantity of PRTC added has fluctuated with time, but most frequently was 150 fmol of PRTC peptides per sample and system suitability injection on nanoflow LC systems. We have found that as low as 15 fmol PRTC/injection is sufficient on most platforms and more cost effective.

In Figure 6, both the system suitability runs and internal QCs in samples suggested the presence of a serious LC-MS system issue. Following a catastrophic turbo pump failure, significant clean-up and troubleshooting of the MS was necessary. Simple calibration of the MS was insufficient - venting the system, disassembling, and cleaning the optics were necessary on several occasions to remove metal debris. Using the longitudinal data from our system suitability runs based on well-characterized pooled sample, we can determine with better confidence what problems necessitate this kind of drastic maintenance rather than a standard re-calibration and heated transfer tube replacement. Finally, the inclusion of the same internal peptide QCs in the experimental samples at equivalent quantities to the system suitability sample is advised to increase the frequency that the system performance is measured. This will enable technicians to more quickly make changes to the system as needed.

The final QCs highlighted in Figure 7 were external QCs. These are two additional samples that are prepared and processed alongside experimental samples within each batch. The inter-batch QC may be used to determine whether sample preparation is reproducible between sample batches and to assess CVs. The inter-experiment QC may be used to assess the validity of the normalization and batch correction pipeline. We previously described the use of these controls with median, Log_2_ normalization and batch adjustment of peptide signal in a recent quantitative proteomics dataset focused on the phenotypic assessment of Alzheimer’s disease in CSF^69^ in additional studies of aging and disease listed on PanoramaWeb in the “Reference Information” section. In recent years, we have most often used median normalization at the peptide-level^74^ but have also examined maxLFQ^75^ and directLFQ^76^ for normalization of peptide and protein-level data. Although originally developed for RNA datasets, we have found, as other groups have indicated, that linear models for microarrays (LIMMA)^77,78^ and ComBat^79,80^ reduce the contribution of technical variation in proteomics datasets. Determination of the optimum normalization and batch correction approach will likely vary according to the sample matrix and experimental approach. The use of external QC samples alongside experimental samples can provide increased confidence in selecting a final analytical approach.

It is important to note that external QCs are not interchangeable with signal calibration. Calibration approaches and their necessary controls have been described by our group and others^81,82^. These approaches may be used in conjunction with these inter-batch and inter-experiment QCs, but the controls described here are not replacements for published calibration techniques.

Naturally, the use of significantly different samples as matrices for external QCs will be of limited utility in assessing sample preparation. To derive the most useful inter-batch QC samples, whenever feasible, equivalent amounts of experimental samples are pooled, aliquoted, and frozen in advance to serve as the inter-batch QC. If pooling small quantities of experimental samples is not feasible, it is advised to collect additional material of the same sample matrix and generate frozen aliquots to be used solely as a control. These pooled aliquots are processed alongside experimental samples in each row of a plate, or once within a batch, and their CVs are calculated to evaluate the reproducibility of the sample preparation within that batch. The inter-experiment QC should be of the same or a very similar matrix to the experimental samples and includes protein and peptide internal QCs. This control should be aliquoted and prepared within each batch either in every row of a plate or once within a batch to assess CV.

While external QC samples are most useful in large-scale sample preparation to identify batch effects and normalize the data, external QC samples can also be useful in situations where experimental plans change unexpectedly. For example, smaller-scale pilot experiments that include external QC samples can be used to later assess how comparable a larger second, third, or fourth batch of similar samples are to one another relative to the initial experiment. Additionally, external QCs are useful in situations where samples of the same matrix or the same type of sample preparation are used regularly in a laboratory. For example, if total plasma isolated with a K_2_-EDTA anticoagulant is regularly considered in different biological settings, it can be helpful to have a large quantity of pooled plasma that has been well-characterized previously on hand as a control. When prepared alongside experimental samples in each new batch or experiment, you can ensure similar performance relative to past projects.

It is strongly recommended that the same internal QCs that were added to the experimental samples are also added to the external QC samples. For example, protein internal QCs can indicate if the external QC sample’s preparation was comparable to others within a batch or across a plate. Peptide internal QCs, such as PRTC, are used to evaluate whether acquisition was consistent.

In closing, we have described a series of real-world applications and case studies of QC measures spanning the assessment of system suitability, sample processing, and data analysis. Moving beyond identifications and relying on a more integrated, adaptable approach to QC in quantitative proteomics can jumpstart troubleshooting LC-MS systems and can increase confidence in the results of experiments.

## Supplementary Material

Supplement 1

## Abbreviations

BSA: bovine serum albumin
CSF: cerebrospinal fluid
DDA: data-dependent acquisition
DIA: data-independent acquisition
DTT: dithiothreitol
ENO: yeast enolase
HCD: high energy collisional dissociation
HPLC: high performance liquid chromatography
IAA: iodoacetamide
LC-MS: liquid chromatography mass spectrometry
MCX: mixed-mode ion exchange
PRTC: peptide retention time calibration mixture
PRM: parallel reaction monitoring
PSM: peptide-spectrum match
PTM: post-translational modification
TEAB: triethylammonium bicarbonate
UPLC: ultra-performance liquid chromatography
