# Supplementary material for: A framework for quality control in quantitative proteomics
Source: bioRxiv. 2024 Apr 25:2024.04.12.589318. Originally published 2024 Apr 13. Preprint. [Version 2] doi: 10.1101/2024.04.12.589318 (PMC11030400; doi:10.1101/2024.04.12.589318)
Supplement: Supplementary file 1 [file NIHPP2024.04.12.589318V2-supplement-1.pdf]

bioRxiv preprint doi: https://doi.org/10.1101/2024.04.12.589318; this version posted April 25, 2024. The copyright holder for this preprint (which was not certified by peer review) is the author/funder, who has granted bioRxiv a license to display the preprint in perpetuity. It is made available under aCC-BY 4.0 International license.

Supplementary Figures

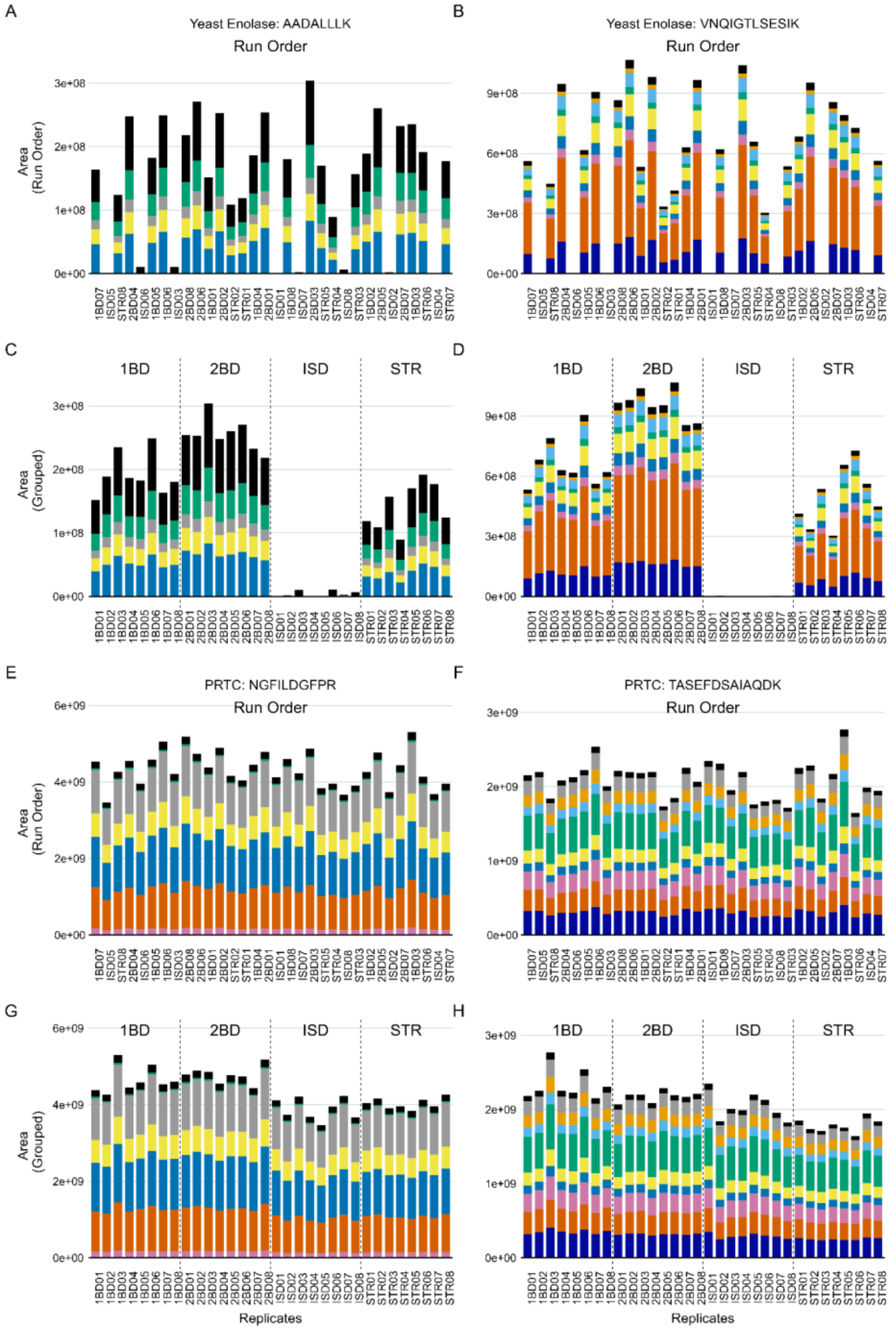

Supplementary Figure 1: The area of two ENO peptides (AADALLLK, VNQIGTLSESIK) and two PRTC peptides (NGFILDGFPR, TASEFDSAIAQDK) representative of observed trends described in Figure 4 are shown here. Peptide VNQIGTLSESIK in panel B and D was also plotted in Figure 4A and 4B. All four peptides are plotted in run order (A, B, E, F) and again grouped by digestion condition (C, D, G, H). ENO peptides were missing in numerous runs (A and B) and when reordered belonged to the ISD sample preparation (C and D). PRTC peptides were broadly consistent across all sample conditions (B, D, F, H). We later found that our RapiGest SF stock, which was one of the only reagents different between the four protocols, was expired and likely impaired the digestion protocol, and thus the ENO, without any impact to the PRTC levels.
